# Importance-Based Key Basic Event Identification and Evolution Mechanism Investigation of Hydraulic Support Failure to Protect Employee Health

**DOI:** 10.3390/s21217240

**Published:** 2021-10-30

**Authors:** Qingwei Xu, Kaili Xu

**Affiliations:** 1 College of Information and Management Science, Henan Agricultural University, Zhengzhou 450046, China; 2 School of Resources and Civil Engineering, Northeastern University, Shenyang 110819, China; xklsafety@163.com

**Keywords:** hydraulic support failure, mining industry, fault tree analysis, chaos theory, synthetic theory model, cause-and-effect-LOPA

## Abstract

Background: Although hydraulic support can help enterprises in their production activities, it can also cause fatal accidents. Methods: This study established a composite risk-assessment method for hydraulic support failure in the mining industry. The key basic event of hydraulic support failure was identified based on fault tree analysis and gray relational analysis, and the evolution mechanism of hydraulic support failure was investigated based on chaos theory, a synthetic theory model, and cause-and-effect-layer-of-protection analysis (LOPA). Results: After the basic events of hydraulic support failure are identified based on fault tree analysis, structure importance (SI), probability importance (PI), critical importance (CI), and Fussell–Vesely importance (FVI) can be calculated. In this study, we proposed the Fussell–Vesely–Xu importance (FVXI) to reflect the comprehensive impact of basic event occurrence and nonoccurrence on the occurrence probability of the top event. Gray relational analysis was introduced to determine the integrated importance (II) of basic events and identify the key basic events. According to chaos theory, hydraulic support failure is the result of cross-coupling and infinite amplification of faults in the employee, object, environment, and management subsystems, and the evolutionary process has an obvious butterfly effect and inherent randomness. With the help of the synthetic theory model, we investigated the social and organizational factors that may lead to hydraulic support failure. The key basic event, jack leakage, was analyzed in depth based on cause-and-effect-LOPA, and corresponding independent protection layers (IPLs) were identified to prevent jack leakage. Implications: The implications of these findings with respect to hydraulic support failure can be regarded as the foundation for accident prevention in practice.

## 1. Introduction

In 2005, the State Council of the People’s Republic of China proposed optimizing the energy structure and vigorously developing clean energy [1]. However, coal is still the main source of energy in China and has made an indelible contribution to economic modernization [2,3]. Coal accounted for 56.8% of the total energy consumption in 2020 in China, according to the National Bureau of Statistics [4]. With the progress of modern mechanized production in the mining industry, various accidents will inevitably occur [5,6,7]. Hydraulic support is a structure used to control the pressure on the coal-mining face and can effectively prevent the gangue from entering the mining face. When the hydraulic support of a coal mine fails, it seriously threatens the lives and safety of miners [8,9,10]. Therefore, a comprehensive analysis of possible failures of hydraulic support is helpful to promote safe practices in coal mines.

Fault tree analysis is a widely used risk-analysis method. Liu et al. investigated risk factors leading to a blowout accident based on fault tree analysis and performed dynamic risk analysis to evaluate the safety of well-control operations [11]. Yazdi and Kabir performed a quantitative risk assessment based on fault tree analysis and identified the most critical events in the fault tree [12]. After determining the basic event of hydraulic support failure based on fault tree analysis, importance analysis can be adopted to identify the key basic event. The importance of the basic event refers mainly to structural importance, probability importance, critical importance, and Fussell–Vesely importance [13,14,15]. Fussell–Vesely importance refers to the impact of basic event nonoccurrence on the occurrence probability of the top event [14]. However, Fussell–Vesely importance cannot determine the comprehensive impact of basic event occurrence and nonoccurrence on the occurrence probability of top events. In this study, a new type of importance was proposed to investigate the comprehensive impact of basic event occurrence and nonoccurrence on the occurrence probability of the top event.

Due to the limitations of human, material, and financial resources, it is impossible to apply the same accident-prevention measures to all basic events. It is necessary to use resources to their best advantage to prevent the occurrence of key basic events. Different types of importance determine different aspects of the impact of basic events on the top event, and it is necessary to determine the integrated impact of different types of importance on the top event. Gray relational analysis is used to measure the degree of correlation between factors based on the degree of similarity in the development trend between factors. Weng et al. proposed a new method to design a recommender system by employing gray relational analysis in heterogeneous social networks [16]. In this study, gray relational analysis was used to determine the key basic event of hydraulic support failure and provides a reference for accident-prevention measures.

The occurrence of accidents has the characteristics of suddenness, complexity, and severity [17,18,19]. Chaos theory investigates mainly the order of behaviors in a system from order to chaos and how to control chaos [20,21,22]. Ding et al. investigated the effect of rotating speeds on running-in quality with the help of chaos theory [21]. Chaos theory is very suitable for exploring the nature of accidents. Unfortunately, previous studies have seldom focused on this issue. In this study, chaos theory was introduced into the field of accident analysis to explore the evolutionary characteristics of hydraulic support failure.

According to Heinrich’s theory of accident causation, accidents are caused mainly by the unsafe state of objects and the unsafe behavior of humans [23,24], both of which are closely related to the social environment. Therefore, the logical relationship between the social environment and accidents should be explored in depth. This study explores the impact of the social environment on the occurrence of accidents with the help of the synthetic theory model [25].

Accident-prevention measures should be taken for the identified key basic events to protect employee health. Frequently used accident-prevention models include the bow tie model [26,27,28] and cause-and-effect-layer-of-protection analysis (LOPA) [29]. Cause-and-effect-LOPA is a composite method that can be used to identify the cause of an accident and take corresponding prevention measures. Xu et al. proposed cause-and-effect-LOPA to investigate the dangerous and harmful factors of foundry accidents in an effort to protect employee health; they identified 19 sub-causes and 18 independent protection layers (IPLs) [29]. To improve the safety level of coal mines, this study seeks to identify prevention measures by identifying key basic events based on cause-and-effect-LOPA.

This study was organized as follows. The fundamental theories and analytical process of the composite risk-assessment approach are summarized in Section 2. The application of the proposed composite risk-assessment approach is illustrated by a case study in Section 3. Discussions of the results and findings are presented in Section 4, and conclusions are presented in Section 5.

## 2. Methods

### 2.1. Framework of This Study

**Figure 1:**
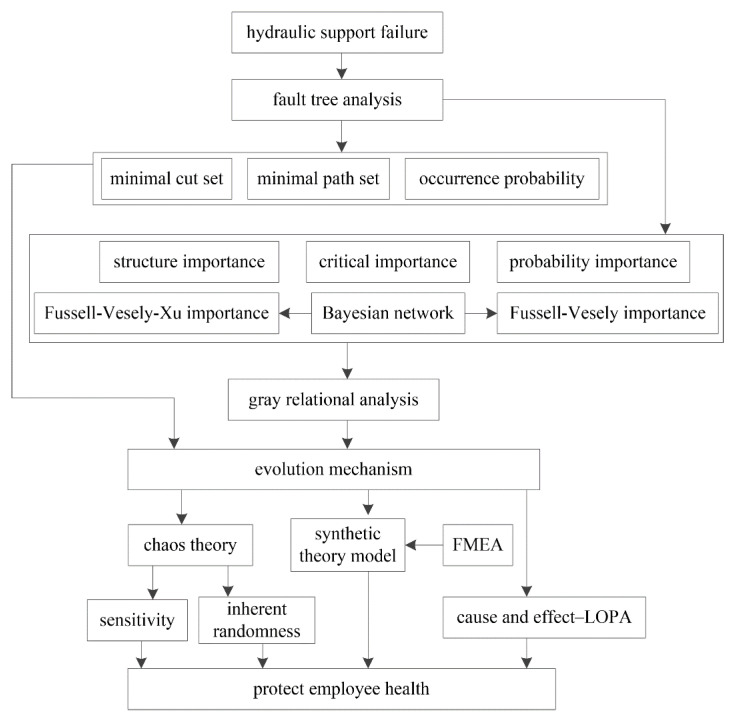
Framework of this study.

The main purpose of this study was to establish a new composite approach to risk assessment, as shown in Figure 1. The basic events of hydraulic support failure can be determined with the help of fault tree analysis [12]. Then, the minimal cut and path sets and the occurrence probability of the top event [11] can be determined. Structure importance (SI) [15], probability importance (PI), critical importance (CI) [30], Fussell–Vesely importance (FVI) [14], and the proposed Fussell–Vesely–Xu importance (FVXI) can be determined based on the structure of the fault tree and the occurrence probability of basic and top events. FVI and FVXI can be calculated with the help of a Bayesian network [31,32,33]. To identify the key basic event, gray relational analysis [16] was introduced to calculate the integrated importance (II) of the basic event. The evolutionary characteristics of hydraulic support failure can be explored by chaos theory [21]. The social and organizational factors of hydraulic support failure were investigated based on the synthetic theory model [25], and the unsafe behaviors of humans were explored by failure mode and effects analysis (FMEA) [34,35]. The prevention measures corresponding to the key basic event can be determined by cause-and-effect-LOPA [29].

Reliability refers to the possibility that the system can work normally. Reliability analysis enables the system reliability to be maximized under certain conditions [36,37]. In other words, reducing the probability of system failure can improve the reliability of the system. After the key basic event of hydraulic support failure is determined based on fault tree analysis [12] and gray relational analysis [16], the reliability of hydraulic support can be improved by reducing the probability of the key basic event. In addition, the influence of the probability of the basic event on the probability of the top event is analyzed in detail in the text.

### 2.2. Fault Tree Analysis

Fault tree analysis builds a logical relationship between atop event and the associated basic events. The approximate occurrence probability of a top event can be calculated by the minimal cut sets, as follows [12]:

(1) $$P\left(T\right)=1-\prod_{\underset{X_{i}\in E_{r}}{r=1}}^{k}\left(1-\prod_{X_{i}\in E_{r}}q_{i}\right)$$

where *E_r_* is the minimal cut set, *q_i_* is the occurrence probability of a basic event, *X_i_*∈*E_r_* denotes the *i*th basic event that belongs to the *r*th minimal cut set, and *k* is the number of minimal cut sets.

#### 2.2.1. Structure Importance

Structure importance assumes the occurrence probability of the basic events is the same and refers to the impact of basic events on the top event based on structure [15]. Structure importance can be calculated as follows:

(2) $$I_{S}\left(i\right)=\frac{1}{k}\sum_{r=1}^{k}\frac{1}{m_{r}}$$

where *m_r_* is the number of basic events of the *r*th minimal cut set.

#### 2.2.2. Probability Importance

Probability importance refers to the impact of the occurrence probability of basic events on the occurrence probability of the top event [30]. Probability importance is a type of Birnbaum importance and can be calculated as follows:

(3) $$I_{B}\left(i\right)=\frac{\partial P\left(T\right)}{\partial q_{i}}$$

#### 2.2.3. Critical Importance

Critical importance refers to the variation rate of the occurrence probability of the top event caused by the variation rate of the occurrence probability of basic events [30]. Critical importance can be calculated as follows:

(4) $$I_{C}\left(i\right)=\underset{\Delta q_{i}\to 0}{\lim}\frac{\Delta P\left(T\right)/P\left(T\right)}{\Delta q_{i}/q_{i}}=\frac{q_{i}}{P\left(T\right)}\cdot I_{B}\left(i\right)$$

#### 2.2.4. Fussell–Vesely Importance

Fussell–Vesely importance is the variation rate of the occurrence probability of thetop event caused by basic event nonoccurrence [14]. Fussell–Vesely importance can be calculated as follows:

(5) $$I_{FV}\left(i\right)=\frac{P\left(T\right)-P\left(T|q_{i}=0\right)}{P\left(T\right)}$$

#### 2.2.5. Fussell–Vesely–Xu Importance

Since Fussell–Vesely importance reflects only the impact of the nonoccurrence of the basic event on the occurrence probability of the top event, we proposed Fussell–Vesely–Xu importance to reflect the impact of both basic event occurrence and nonoccurrence on the occurrence probability of the top event. Fussell–Vesely–Xu importance can be calculated as follows:

(6) $$I_{FV}\left(i\right)=\frac{P\left(T|q_{i}=1\right)-P\left(T|q_{i}=0\right)}{P\left(T\right)}$$

### 2.3. Bayesian Network

The Bayesian network includes network nodes, directed links, conditional probabilities of nodes, and a directed acyclic graph and can reflect uncertain relationships among network nodes [31,32,33]. The Bayesian network is based on the Bayesian formula, and the probability of event *A* under the occurrence of event *B* can be expressed as follows:

(7) $$P\left(A|B\right)=\frac{P\left(B|A\right)\times P\left(A\right)}{P\left(B\right)}$$

where *P*(*A*) is the prior probability of event *A*,*P*(*A*|*B*) is the posterior probability of event *A* under the occurrence of event *B*,*P*(*B*|*A*) is the conditional probability of event *B* under the occurrence of event *A*,*P*(*B*) is the prior probability of event *B*,*P*(*A*) is not related to event *B*, and *P*(*B*) is not associated with event *A*.

Let the set of events *A* be $A=\left\{a_{1},a_{2},\cdots ,a_{n}\right\}$. Then, the Bayesian formula of *P*(*B*) can be expressed as follows:

(8) $$P\left(B\right)=\sum_{i=1}^{n}P\left(B|a_{i}\right)P\left(a_{i}\right)$$

The occurrence probability of a specific accident can be derived by the prior probability of basic events with a Bayesian network, and the Bayesian network reflects the relationship between prior and posterior probability.

### 2.4. Gray Relational Analysis

Gray relational analysis is used to identify the optimal item by calculating the gray relational degree between the ideal item and the given items [16]. The procedure of gray relational analysis is as follows.

Let the data of given items be *A* = [*a_ij_*], where *a_ij_* is the original data of the *j*th evaluation indicator of the *i*th given item, *m* is the number of given items, and *n* is the number of evaluation indicators. The matrix *B* = [*b_j_*] is the ideal item, where *b_j_* is the ideal value of the *j*th evaluation indicator.

Let the ideal item, *B*, be the reference sequence and given items, *A*, be the sequences to be compared. The gray relational coefficient of the *j*th evaluation indicator of the *i*th given item can then be calculated as follows:

(9) ξij=min1≤i≤m1≤j≤n|bj−aij|+max1≤i≤m1≤j≤n|bj−aij|2|bj−aij|+max1≤i≤m1≤j≤n|bj−aij|2

Let the weights of the evaluation indicators be *W* = [*w*_1_, *w*_2_, …, *w_n_*]; then, the gray relational degree of the given items can be determined as follows:

(10) $$r_{i}=\sum_{j=1}^{n}\xi_{ij}\times w_{j}\;\;\;i=1,2,\cdots ,m$$

The larger the gray relational degree, the closer the given item is to the ideal item. The order of the given items can be determined by this process to successfully identify the optimal item.

### 2.5. Cause-and-Effect-LOPA

**Figure 2:**
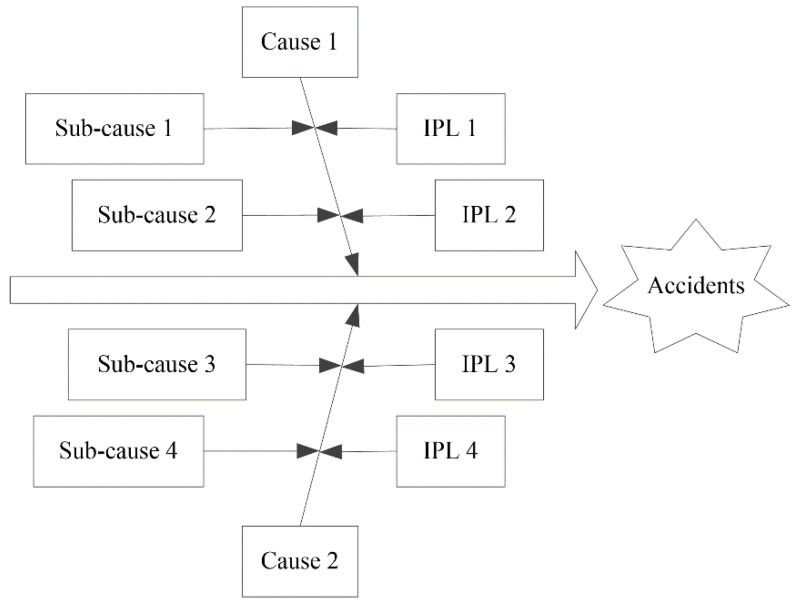
Cause-and-effect-LOPA diagram.

Once the safety level of the casting workshop is achieved, corresponding safety measures should be adopted. Cause-and-effect-LOPA [29] identifies factors that may lead to accidents and describes IPLs that could be applied to prevent accidents (Figure 2).

## 3. Results

### 3.1. Fault Tree Analysis of Hydraulic Support Failure

**Figure 3:**
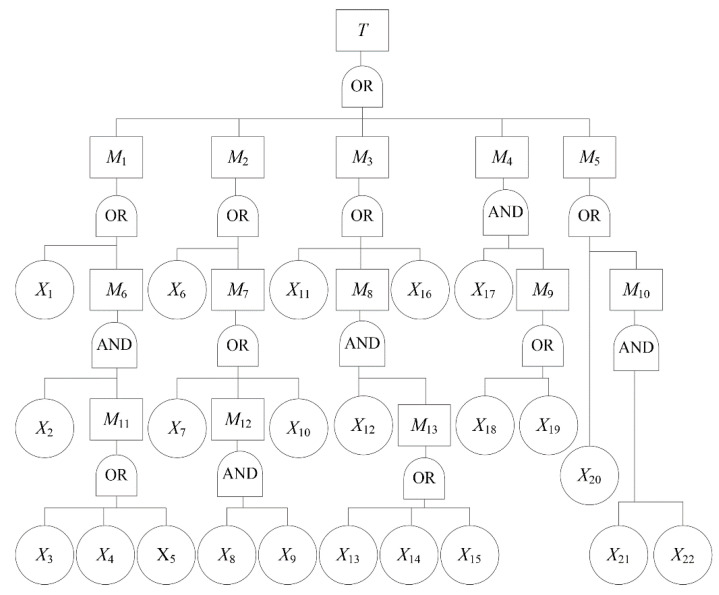
Fault tree of hydraulic support failure.

According to the production practices of coal mines, a fault tree of hydraulic support failure can be described as shown in Figure 3 [38].In Figure 3, *T* denotes the top event, namely hydraulic support failure; *M*_1_ denotes operating valve failure; *M*_2_ denotes upright post failure; *M*_3_ denotes jack failure; *M*_4_ denotes pedestal failure; *M*_5_ denotes emulsion pump failure; *M*_6_ denotes handle failure; *M*_7_ denotes safety valve failure; *M*_8_ denotes pipeline failure; *M*_9_ denotes a pedestal break; *M*_10_ denotes unqualified emulsion; *M*_11_ denotes an inflexible handle that cannot self-lock; *M*_12_ denotes safety valve leakage; *M*_13_ denotes a pipeline defect; *X*_1_ denotes leakage outside the valve; *X*_2_ denotes that the handle was not checked carefully; *X*_3_ denotes that the swinging angle of the handle is less than 80°; *X*_4_ denotes work supervisor fatigue; *X*_5_ denotes too much coal dust on the handle; *X*_6_ denotes upright post deformation; *X*_7_ denotes low safety valve pressure; *X*_8_ denotes poor O-ring inspection; *X*_9_ denotes a damaged O-ring seal; *X*_10_ denotes spring failure; *X*_11_ denotes jack deformation; *X*_12_ denotes inadequate pipeline inspection; *X*_13_ denotes high-pressure flexible pipe leakage; *X*_14_ denotes a damaged flexible pipe connector; *X*_15_ denotes pipeline blockage; *X*_16_ denotes jack leakage; *X*_17_ denotes failure to find a break in time; *X*_18_ denotes the main reinforcement break; *X*_19_ denotes a ball-and-socket break; *X*_20_ denotes insufficient pump pressure; *X*_21_ denotes failure to test oil; and *X*_22_ denotes polluted emulsion.

In the fault tree of hydraulic support failure, there are a total of 14 logic gates: nine logic OR gates and five logic AND gates. Logic OR gates account for 64%, which shows that the occurrence probability of a top event is greater; that is, the occurrence probability of hydraulic support failure in the coal mine is high.

According to the fault tree of hydraulic support failure, the structural equation can be obtained as follows:

*T* = *M*_1_+ *M*_2_+ *M*_3_+ *M*_4_+ *M*_5_

There are 17 minimal cut sets in the fault tree of hydraulic support failure, according to the Boolean algebra algorithm [39], as shown in Table S1. The minimal cut set indicates the possible path of the top event; that is, there are 17 paths that may lead to hydraulic support failure.

There are 32 minimal path sets in the fault tree of hydraulic support failure, according to the Boolean algebra algorithm [39], as shown in Table S2. The minimal path set indicates possible ways to prevent the occurrence of the top event; that is, there are 32 paths that can be adopted to prevent the occurrence of hydraulic support failure. However, there are many basic events in each minimal path set; thus, preventing all the basic events in a minimal path set will be difficult.

To obtain the occurrence probability of hydraulic support failure, we must first determine the occurrence probability of each basic event. The occurrence probability of each basic event is shown in Table 1.

The occurrence probability of hydraulic support failure can be achieved based on Equation (1), and the result is *P*(*T*) = 0.066273.

### 3.2. Importance of Basic Events

The SI, PI, and CI of basic events can be calculated based on Equations (2)–(4), and the results are shown in Table 2.

To determine FVI, it is necessary to calculate the occurrence probability of hydraulic support failure based on basic event nonoccurrence, namely *P*(*T*|*x*_i_ = 0). *P*(*T*|*x*_i_ = 0) can be determined with the help of the Bayesian network.

**Figure 4:**
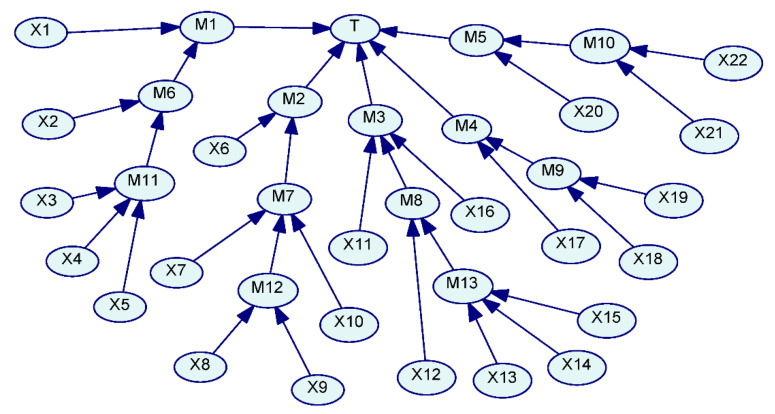
Bayesian network of hydraulic support failure.

The fault tree of hydraulic support failure can be transferred into the Bayesian network, as shown in Figure 4.

With the help of the forward reasoning ability of the Bayesian network, assuming that the basic event of hydraulic support failure does not occur, the occurrence probability of hydraulic support failure can be obtained in this case; that is, *P*(*T*|*x*_i_ = 0), as shown in Table 1.

The FVI of basic events can be calculated based on Equation (5), as shown in Table 2.

The FVXI of basic events can be calculated based on Equation (6), as shown in Table 2.

### 3.3. II Based on Gray Relational Analysis

Since different types of importance reflect the different properties of basic events, the importance rankings of the different basic events differ in Table 1. Gray relational analysis is used to determine the ranking of different indicators by calculating the gray relational degree [16]. Therefore, gray relational analysis was adopted to calculate the II of basic events.

The data matrix of the importance ranking of the basic events of hydraulic support failure is shown below.

$$A={\left[\begin{array}{l} 2\;\;\;1\;\;\;\;3\;\;\;\;\;3\;\;\;\;\;3\;\;\;2\;\;\;2\;\;\;3\;\;\;\;3\;\;\;\;2\;\;\;2\;\;\;1\;\;\;\;3\;\;\;\;3\;\;\;\;\;3\;\;\;\;2\;\;\;2\;\;\;\;3\;\;\;\;\;3\;\;\;\;2\;\;\;3\;\;\;\;3\\ 2\;\;\;8\;\;\;10\;\;\;10\;\;\;10\;\;\;6\;\;\;3\;\;\;9\;\;\;10\;\;\;4\;\;\;5\;\;\;7\;\;\;10\;\;\;10\;\;\;10\;\;\;1\;\;\;11\;\;\;10\;\;\;10\;\;\;4\;\;\;9\;\;\;10\\ 2\;\;\;8\;\;\;10\;\;\;10\;\;\;10\;\;\;9\;\;\;3\;\;\;9\;\;\;\;9\;\;\;4\;\;\;5\;\;\;6\;\;\;\;7\;\;\;\;9\;\;\;\;\;9\;\;\;\;1\;\;\;11\;\;\;12\;\;\;12\;\;\;4\;\;\;9\;\;\;9\\ 2\;\;\;8\;\;\;9\;\;\;\;\;9\;\;\;\;9\;\;\;\;8\;\;\;3\;\;\;8\;\;\;\;8\;\;\;\;4\;\;\;5\;\;\;6\;\;\;\;7\;\;\;\;8\;\;\;\;\;8\;\;\;\;1\;\;\;\;9\;\;\;\;9\;\;\;\;\;9\;\;\;\;4\;\;\;8\;\;\;8\\ 2\;\;\;8\;\;\;11\;\;\;11\;\;\;11\;\;\;6\;\;\;3\;\;\;9\;\;\;10\;\;\;4\;\;\;5\;\;\;7\;\;\;12\;\;\;13\;\;\;13\;\;\;1\;\;\;14\;\;\;10\;\;\;10\;\;\;\;4\;\;\;9\;\;\;10 \end{array}\right]}^{’}$$

In matrix *A*, the rows from top to bottom indicate the importance rankings of SI, PI, CI, FVI, and FVXI, and the columns from left to right indicate the importance rankings of basic events from *X*_1_ to *X*_22_.The optimal matrix of the basic event is *B* = [1 1 1 1 1]. Since different types of importance reflect the different properties of basic events, we cannot determine which type is most important. Therefore, the importance is assigned the same weight; that is, *W* = [0.2 0.2 0.2 0.2 0.2].

The II of basic events can be calculated based on Equations (9)–(10), as shown in Table 2.

Table 2 shows that the II of basic event *X*_16_ is maximal. That is, basic event *X*_16_ contributes the most to the occurrence of hydraulic support failure.

To test the reliability of the proposed method, contrastive analysis was carried out. The II results indicate that basic event *X*_16_ contributes the most to hydraulic support failure and that basic event *X*_1_ ranks second. Then, we investigated the influence of the rate of change in the probability of these basic events on the probability of the top event, as shown in Table 3.

As shown in Table 3, the probability of the top event changes with the probability of each basic event, but basic event *X*_16_ has a more severe impact on the probability of the top event, which is in line with the results of the importance analysis. Reducing the probability of basic event *X*_16_ will significantly improve the reliability of hydraulic support.

This study proposed a new method to identify the key basic event in relation to the top event. Each enterprise has limited manpower and material and financial resources. It is impossible for enterprises to allocate the same safety input to every hazard factor. To ensure safe production and reduce costs, more safety input is designated for more hazard factors. The II determined by gray relational analysis can identify the key basic event more accurately than other methods, thus reducing blindness to safety input.

Vaurio [40] described the calculation method for basic event importance to the top event. Zhu et al. [15] calculated the basic event importance of the top event and determined the critical basic event by comparing the numerical value of each type of importance of basic events. Different types of importance are used to examine different aspects of the impact of basic events on the top event. For the same top event, the ranking of basic events obtained by different importance calculation methods also differs. To identify the key basic event, this study proposed II based on gray relational analysis, which compensates for the shortcomings of previous studies [38].

### 3.4. Evaluation Mechanism of Hydraulic Support Failure

#### 3.4.1. Chaotic Characteristics in the Evaluation Process of Hydraulic Support Failure

(1) The sensitivity of the evolutionary process to initial conditions

**Figure 5:**
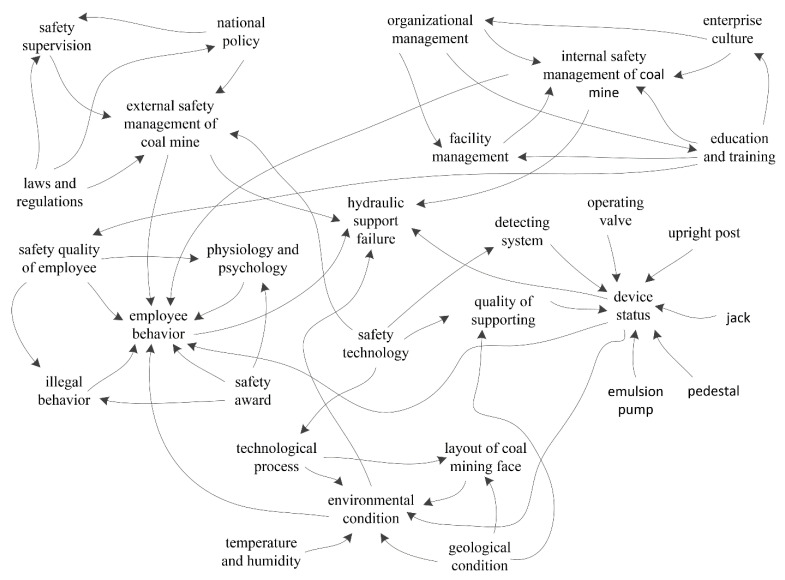
Butterfly effect in the evolutionary process of hydraulic support failure.

The hydraulic support failure process is affected by the cross-coupling of the employee, object, environment, and management subsystems, and a fault in any link of the system may cause changes in the state of each subsystem. When this change disrupts the balance of a subsystem, hydraulic support failure may occur, as shown in Figure 5.

In Figure 5, the direction of an arrow indicates that one factor has an influence on another factor. The butterfly effect means that, when a butterfly in the tropical rainforest of the Amazon River in South America flaps its wings, it may cause a tornado in Texas, USA, and even a slight deviation in the butterfly’s flapping wings will change the direction of the tornado [41].

According to chaos theory, hydraulic support failure is the result of the cross-coupling and infinite amplification of faults in the employee, object, environment, and management subsystems, and its evolutionary process has an obvious butterfly effect (Figure 5). For example, a hydraulic support failure accident occurred in the Shenghua coal mine [9]. The link faults in the system that led to hydraulic support failure accident included the following aspects. First, the main roof broke in front of the working face. Second, the strata became unstable. Third, these changes induced a sharp load increase on the hydraulic support. Last, the hydraulic support failure accident occurred. These faults were amplified after cross-coupling and eventually led to the hydraulic support failure, reflecting the butterfly effect in the chaos theory of the evolutionary process of hydraulic support failure (Figure 5).

In a nonlinear system, small errors in a certain factor are not always small. Under appropriate conditions, such small errors will evolve and develop infinitely, leading to consequences for the system that are difficult to estimate. According to chaos theory, a small input error in the system can cause a substantial drift in output under certain conditions in a nonlinear system. In the actual production process, since the system will inevitably be disturbed by external factors, a small error at the initial moment will be amplified over time, leading to unpredictable consequences.

The direct causes of the top event can be identified based on fault tree analysis [11]. Fault tree analysis focuses mainly on the hazard factors that may lead to the occurrence of the top event. However, it is difficult for the fault tee to effectively analyze in-depth factors, such as management and environmental factors [38]. With the help of chaos theory; the human, object, environment, and management factors; and their interactions can all be identified, compensating for the shortcomings of fault tree analysis [38].

(2) The inherent randomness of the evolutionary process

The occurrence of hydraulic support failure requires two conditions: periodic pressure and the failure of the cylinder stroke of hydraulic support. The roof pressure of the working face is relieved through the safety valve in a timely manner under normal circumstances. However, dynamic changes in each subsystem may randomly affect the cylinder stroke of hydraulic support. On the one hand, the hydraulic support system can bear such pressure for a short period, prompting the working resistance of the upright post to increase rapidly until it reaches or exceeds the rated setting force; the safety valve then releases the load to maintain balance. However, when the hydraulic support safety valve discharges frequently, the stroke of the upright post decreases, eventually causing the hydraulic support cylinder stroke to fail. On the other hand, with developments in science and technology, the degree of mechanization and automation of production has been greatly improved. However, the safety knowledge and professional skills of employees may not match the degree of mechanization and automation of the production process, which may cause random hydraulic support accidents owing to such issues as illegal operation.

#### 3.4.2. Synthetic Theory Model of Hydraulic Support Failure

**Figure 6:**
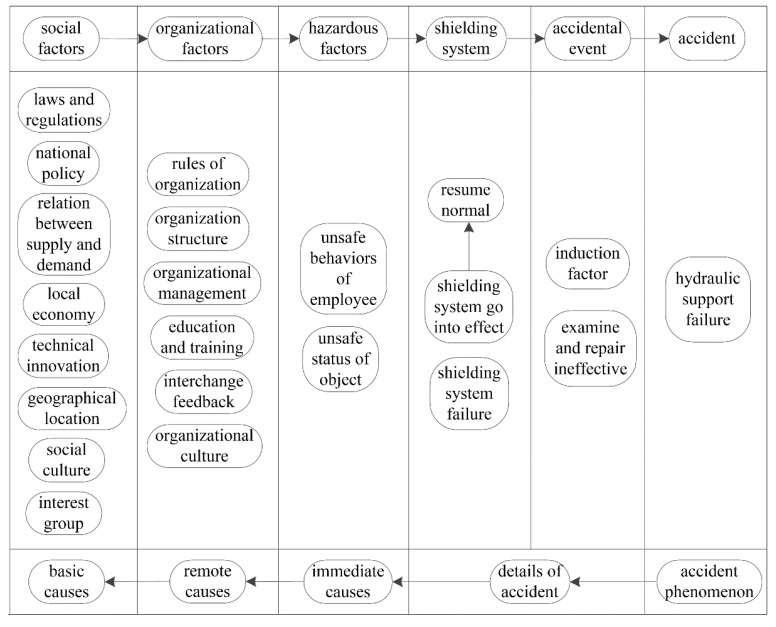
Synthetic theory model of hydraulic support failure.

The organizational errors of hydraulic support failure are initially caused by social factors, and the hazard factors are then triggered by accident factors associated with employees and objects. Hydraulic support failure is the result of the comprehensive effects of internal and external coal-mine factors. The synthetic theory model of hydraulic support failure is shown in Figure 6.

According to accident causation theory, accidents caused by unsafe behaviors by humans account for approximately 90% of all accidents [24]. Therefore, reducing the unsafe behaviors of humans is of great significance for ensuring enterprise safety. Then, FMEA can be adopted to analyze hydraulic support failure and to indicate how to control the unsafe behaviors of humans, as shown in Table 4.

As shown in Table 4, the modes, reasons, and effects of hydraulic support failure and the unsafe behaviors of employees can be identified based on FMEA. Moreover, countermeasures are presented to prevent hydraulic support failure and reduce the unsafe behaviors of employees. Through FMEA, the unsafe behaviors of employees can be reduced, and the reliability of hydraulic support can be improved.

The unsafe status of objects is a major cause of accidents, according to accident causality theory [24]. Many pieces of equipment are used in the production process of an enterprise, and the status of the equipment is of great significance in ensuring safe production. Understanding the legal requirements and regulations for the use of equipment can enable enterprises and employees to better carry out safe production activities.

The *Safe Production Law* is a special law regarding production safety in China [42]. The main purpose of the *Safety Production Law* is to strengthen the supervision and administration of production safety to prevent and reduce accidents, to protect people’s lives and property safety, and to promote economic development. The *Safe Production Law* has detailed regulations for the use of equipment, which are as follows. When using new equipment, enterprises must understand and master its safety technical characteristics, implement effective safety precautions, and conduct special safety education and training for employees. Enterprises should install obvious safety warning signs on equipment with hazard factors. The design, manufacture, installation, use, testing, maintenance, transformation, and disposal of equipment should comply with national standards. Enterprises must conduct regular maintenance and testing of equipment to ensure normal operation. The state has implemented an elimination system for equipment that seriously endangers production.

One of the purposes of the *Labor Law* is to protect the legitimate rights and interests of employees [43]. If equipment that affects production and public interests breaks down, it must be repaired in time based on the *Labor Law*.

To ensure safe production, enterprises should strictly adhere to the laws and regulations.

#### 3.4.3. Cause-and-Effect-LOPA of Basic Event X_16_

**Figure 7:**
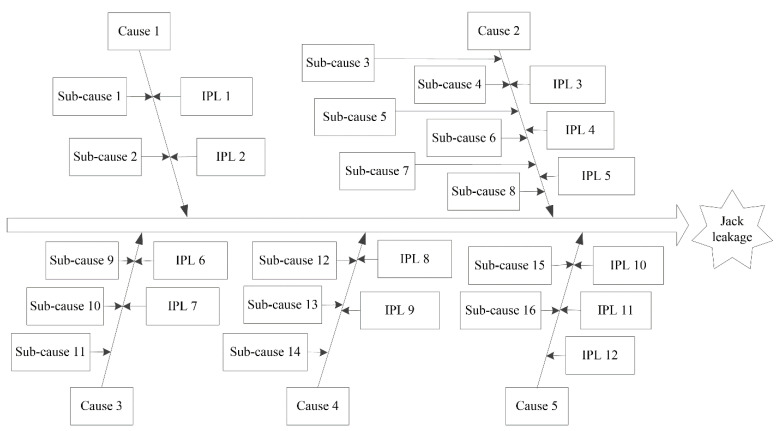
Cause-and-effect-LOPA of jack leakage.

The basic event *X*_16_, jack leakage, is the key basic event according to the importance analysis. Controlling hazard factors helps to promote the safe operation of coal mining enterprises. To identify hazard factors that cause jack leakage and take corresponding preventive measures, cause-and-effect-LOPA on jack leakage was carried out, as shown in Figure 7.

There are five reasons for jack leakage, and these five reasons can be further subdivided into 16 subcategories, as shown in Table 5.

The preventive measures that should be taken to prevent the jack from leaking are shown in Table 6.

In this cause-and-effect-LOPA, five causes and 16 sub-causes may lead to jack leakage, and jack leakage can be prevented by 12 IPLs. The performance of hydraulic support can be improved by preventing the jack from leaking.

## 4. Discussion

### 4.1. Comparison with Previous Studies

Fussell–Vesely importance can be used to investigate the impact of basic event nonoccurrence on the occurrence probability of the top event [14]. However, Fussell–Vesely importance cannot determine the comprehensive impact of the basic event occurrence and nonoccurrence on the occurrence probability of the top event. This study proposed Fussell–Vesely–Xu importance, which can simultaneously consider the comprehensive impact of basic event occurrence and nonoccurrence on the occurrence probability of the top event. Fussell–Vesely–Xuimportance can be calculated with the help of a Bayesian network [31,32,33], which reduces the difficulty of calculation. Fussell–Vesely–Xu importance expands the ability to analyze the importance of the fault tree.

Each type of importance applies to different aspects of the influence of basic events on the occurrence probability of the top event [14]. Due to the limitations of human, material, and financial resources, it is impossible to apply the same accident-prevention measures to all basic events. Ranking the importance of basic events may cause confusion for decision-makers. Unfortunately, previous studies have not provided an appropriate method for determining the comprehensive importance of basic events [14,38]. In this study, gray relational analysis [16] was introduced to calculate the II of basic events and to provide a reference for decision-makers to implement targeted prevention measures.

Fault tree analysis is used to identify the basic events based on possible accidents or accidents that have occurred in the system [11]. However, hydraulic support failure is a very complex phenomenon, and its occurrence is random and cannot be completely determined by basic events. The occurrence of an accident is comprehensively affected by human, machine, and environmental factors [15]. There are internal relations among these factors, and an abnormality in a factor may cause hydraulic support failure. Additionally, the evolutionary process has an obvious butterfly effect (Figure 5). Mao and Liu [38] investigated only the basic events of hydraulic support failure and failed to explore their interrelationships. In this study, chaos theory was introduced into the analysis of hydraulic support failure to help improve our understanding of the complex characteristics of accidents in coal mines.

According to Heinrich’s theory of accident causation, accidents are caused mainly by the unsafe state of the object and the unsafe behavior of humans [24]. The unsafe state of the object and the unsafe behavior of humans are only the external factors that lead to the accident, while social factors and organizational factors are the underlying causes (Figure 6). To fundamentally prevent hydraulic support failure in coal mines, it is necessary to gradually improve the social and organizational factors. Previous studies on accident causation theory have focused mainly on human and machine factors [23,24]. In this study, a synthetic theory model was adopted to investigate the social and organizational factors of accidents, which is helpful in fully understanding the causes of accidents.

Fault tree analysis has performed well in identifying the causes of accidents, but it is insufficient in terms of accident-prevention measures [11,12]. Mao and Liu [38] identified a combination of causes of hydraulic support failure based on fault tree analysis, that is, the minimal cut set. What kind of prevention measures should be taken to prevent the occurrence of causes in the minimal cut set? Although the fault tree analysis indicates the direction, it does not have the corresponding processing function. Cause-and-effect analysis cannot determine prevention measures for accident causes and is not effective in preventing accidents [44]. LOPA is a semi-quantitative assessment method of accident scenarios that analyses the initiating event, consequences, and IPL [45]. Cause-and-effect-LOPA is a composite method that can identify the cause of an accident and determine the corresponding prevention measures. The results show that cause-and-effect-LOPA can determine prevention measures for identified accident causes and significantly reduce the risk of an accident (Figure 7).

### 4.2. Implications

The direct causes of hydraulic support failure are mainly the unsafe behaviors of employees and the unsafe status of objects based on synthetic theory analysis (Figure 6). The main unsafe behaviors of employees are disobeying rules, operating in violation of rules, and artificial operational errors. The unsafe status of objects refers mainly to incomplete or defective safety protection equipment, production activities under severe environmental conditions, and production activities under severe geological conditions.

To prevent the unsafe behaviors of employees, the following prevention measures should be adopted: formulating operating procedures for production activities, punishing those who violate these procedures, conducting regular employee health checks, and increasing the education and training for employees. Employees should wear the necessary safety protection equipment. In addition, the frequency of work-site safety inspections should be increased, and violations should be addressed in a timely manner.

To prevent unsafe object status, the following prevention measures should be adopted: employees should use safety protection equipment based on operational requirements and check the status of safety protection equipment. Production specifications should be formulated for abnormal conditions, and production activities under severe conditions should be prohibited. The environmental conditions required for on-site operations should be specified. For the geological structure zone, the range of influence should be first determined, and countermeasures should then be proposed.

Laws and regulations also have an important influence on the safe production of coal mines, according to chaos theory (Figure 5). If an enterprise does not comply with laws and regulations, hydraulic support failure may occur. During production activities, the enterprise should protect employees’ lives and safety and adhere to the concept of safety first. To resolve hazard factors at the source, comprehensive measures should be adopted. The enterprise should strictly abide by the provisions of laws and regulations.

### 4.3. Limitations

To simplify the discussion, the weights of the importance of all different basic events were set at the same level. If the weights of the importance of different basic events were set at different levels, the ranking of the importance of basic events may also differ. Future studies should focus on the influence of the weights of importance on the II of basic events. For example, we could invite experts, such as university teachers and students, enterprise managers, and employees engaged in production safety, to rate each type of importance of basic events. According to the expert scores, the analytic hierarchy process [46] would then be adopted to determine the weight of each type of importance, and a consistency test should also be carried out.

## 5. Conclusions

This study proposed a composite risk-assessment method for hydraulic support failure in the mining industry. The main conclusions are presented below.

Hydraulic support failure is the result of the cross-coupling and infinite amplification of faults in the employee, object, environment, and management subsystems, and its evolutionary process has an obvious butterfly effect. If hazard factors are triggered by social and organizational factors, then hydraulic support failure may occur. The unsafe behaviors of employees and the unsafe status of objects are the direct causes of hydraulic support failure; organizational factors are the remote causes, and social factors are the basic causes. For hydraulic support failure in this study, jack leakage is the key basic event. To prevent jack leakage, 12 IPLs were adopted. Through the method used in this study, the risk of hydraulic support failure can be greatly reduced.

## Figures and Tables

**Table 1:** Probability of basic events and top event.

Basic Event	Occurrence Probability	*P*(*T*|*x*_i_ = 1)	*P*(*T*|*x*_i_ = 0)
*X*_1_	0.01	1	0.05684
*X*_2_	0.001	0.0802	0.06626
*X*_3_	0.005	0.06719	0.06627
*X*_4_	0.005	0.06719	0.06627
*X*_5_	0.005	0.06719	0.06627
*X*_6_	0.00001	1	0.06626
*X*_7_	0.005	1	0.06158
*X*_8_	0.001	0.0756	0.06626
*X*_9_	0.01	0.0672	0.06626
*X*_10_	0.001	1	0.06534
*X*_11_	0.0001	1	0.06618
*X*_12_	0.001	0.13055	0.06621
*X*_13_	0.05	0.06714	0.06623
*X*_14_	0.01	0.06714	0.06626
*X*_15_	0.01	0.06714	0.06626
*X*_16_	0.05	1	0.01713
*X*_17_	0.001	0.06646	0.06627
*X*_18_	0.0001	0.06721	0.06627
*X*_19_	0.0001	0.06721	0.06627
*X*_20_	0.001	1	0.06534
*X*_21_	0.001	0.0756	0.06626
*X*_22_	0.01	0.0672	0.06626

**Table 2:** Importance of basic events of hydraulic support failure.

Basic Event	SI		PI		CI		FVI		FVXI		II	
	Value	R	Value	R	Value	R	Value	R	Value	R	Value	R
*X*_1_	0.0588	2	0.943157	2	0.142312	2	0.142348	2	14.2312	2	0.8667	2
*X*_2_	0.1765	1	0.014006	8	0.000211	8	0.000211	8	0.2103	8	0.5852	8
*X*_3_	0.0294	3	0.000934	10	0.00007	10	0.00006	9	0.0139	11	0.4891	13
*X*_4_	0.0294	3	0.000934	10	0.00007	10	0.00006	9	0.0139	11	0.4891	13
*X*_5_	0.0294	3	0.000934	10	0.00007	10	0.00006	9	0.0139	11	0.4891	13
*X*_6_	0.0588	2	0.933735	6	0.000141	9	0.000211	8	14.0891	6	0.5854	7
*X*_7_	0.0588	2	0.938418	3	0.070798	3	0.070827	3	14.1597	3	0.7851	3
*X*_8_	0.0294	3	0.009337	9	0.000141	9	0.000211	8	0.1409	9	0.5182	10
*X*_9_	0.0294	3	0.000934	10	0.000141	9	0.000211	8	0.0142	10	0.5066	11
*X*_10_	0.0588	2	0.934661	4	0.014103	4	0.014093	4	14.103	4	0.7207	4
*X*_11_	0.0588	2	0.933819	5	0.001409	5	0.001418	5	14.0903	5	0.6686	5
*X*_12_	0.1765	1	0.065363	7	0.000986	6	0.000966	6	0.9708	7	0.6341	6
*X*_13_	0.0294	3	0.000934	10	0.000705	7	0.000664	7	0.0137	12	0.5191	9
*X*_14_	0.0294	3	0.000934	10	0.000141	9	0.000211	8	0.0133	13	0.493	12
*X*_15_	0.0294	3	0.000934	10	0.000141	9	0.000211	8	0.0133	13	0.493	12
*X*_16_	0.0588	2	0.982869	1	0.741520	1	0.741528	1	14.8304	1	0.9733	1
*X*_17_	0.0588	2	0.000187	11	0.000003	11	0.00006	9	0.0029	14	0.4872	14
*X*_18_	0.0294	3	0.000934	10	0.000001	12	0.00006	9	0.0142	10	0.4846	15
*X*_19_	0.0294	3	0.000934	10	0.000001	12	0.00006	9	0.0142	10	0.4846	15
*X*_20_	0.0588	2	0.934661	4	0.014103	4	0.014093	4	14.103	4	0.7207	4
*X*_21_	0.0294	3	0.009337	9	0.000141	9	0.000211	8	0.1409	9	0.5182	10
*X*_22_	0.0294	3	0.000934	10	0.000141	9	0.000211	8	0.0142	10	0.5066	11

Note: R is ranking.

**Table 3:** Influence of probability of basic event on probability of top event.

*X*_16_	*P*(*T*)	*X*_16_	*P*(*T*)	*X*_1_	*P*(*T*)	*X*_1_	*P*(*T*)
+10%	+7.41%	−10%	−7.41%	+10%	+1.42%	−10%	−1.42%
+20%	+14.83%	−20%	−14.83%	+20%	+2.85%	−20%	−2.85%
+30%	+22.25%	−30%	−22.25%	+30%	+4.27%	−30%	−4.27%
+40%	+29.66%	−40%	−29.66%	+40%	+5.69%	−40%	−5.69%
+50%	+37.08%	−50%	−37.08%	+50%	+7.12%	−50%	−7.12%

**Table 4:** FMEA of hydraulic support failure.

Subsystem	Failure Modes	Failure Reasons	Failure Effects	Countermeasures
Connector of flexible pipe	Breakdown	Connector of flexible pipe falls off Connector of flexible pipe is not tightly crimped Seal connector of flexible pipe is damaged Connector of flexible pipe is blocked	No oil pressure in pipeline system No action in operation of pipeline system	Fasten flexible pipe connector Replace seal connector of flexible pipe Straighten flexible pipe connector Replace flexible pipe connector
Employee	Mis-operation	Unfamiliar with operational skills Reduced equipment sensitivity Employee is emotional Environmental factors	Hydraulic support failure Accident with casualties	Strengthen education and training Overhaul equipment in a timely manner Keep employees in a stable state at work Improve on-site working conditions

**Table 5:** Causes of jack leakage.

Cause	Description	Cause	Description
Cause 1	Unreasonable design	Sub-cause 7	Surface defects of seals
Cause 2	Quality of seal is substandard	Sub-cause 8	Poor storage environment for seals
Cause 3	Processing technology	Sub-cause 9	Coaxiality error between components
Cause 4	Assembly process	Sub-cause 10	Improper processing of oversealing chamfering
Cause 5	On-site usage	Sub-cause 11	Improper processing of sealing fillets
Sub-cause 1	Inappropriate fit clearance between moving parts	Sub-cause 12	Dust between components
Sub-cause 2	Improper surface roughness of sealing groove	Sub-cause 13	Sharp burrs between components
Sub-cause 3	Poor wear resistance of seals	Sub-cause 14	Damage to sealing lip
Sub-cause 4	Poor surface stability of seals	Sub-cause 15	Hard object percussion
Sub-cause 5	Poor hydrolysis resistance of seals	Sub-cause 16	Bump in coating of piston rod
Sub-cause 6	Large dimensional tolerance of seals		

**Table 6:** Countermeasures to prevent the jack from leaking.

IPL	Description	IPL	Description
IPL 1	Improve product design	IPL 7	Strictly follow processing technology for manufacturing
IPL 2	Strengthen knowledge training for designers	IPL 8	Strictly clean parts before assembly
IPL 3	Choose high-quality seal materials	IPL 9	Use special tools to assemble seals
IPL 4	Improve storage environment of seals	IPL 10	Choose appropriate emulsifier
IPL 5	Optimize manufacturing process of seals	IPL 11	Replace emulsion in time
IPL 6	Optimize processing technology of parts	IPL 12	Keep piping system clean

## Data Availability

All relevant data are within the paper.

## Supplementary Materials

The following are available online at https://www.mdpi.com/article/10.3390/s21217240/s1, Table S1: The minimal cut sets of hydraulic support failure; Table S2: The minimal path sets of hydraulic support failure.
