# Supplementary material for: Importance-Based Key Basic Event Identification and Evolution Mechanism Investigation of Hydraulic Support Failure to Protect Employee Health
Source: Sensors (Basel). 2021 Oct 30;21(21):7240. doi: 10.3390/s21217240 (PMC8587061; doi:10.3390/s21217240)
Supplement: Supplementary file 1 [file sensors-21-07240-s001.zip › sensors-1396411-supplementary.pdf]

Table S1. The minimal cut sets of hydraulic support failure

**Minimal cut set**	**Basic event**
C_1	X_1
C_2	X_{10}
C_3	X_{11}
C_4	X_{12}, X_{13}
C_5	X_{12}, X_{14}
C_6	X_{12}, X_{15}
C_7	X_{16}
C_8	X_{17}, X_{18}
C_9	X_{17}, X_{19}
C_{10}	X_2, X_3
C_{11}	X_2, X_4
C_{12}	X_2, X_5
C_{13}	X_{20}
C_{14}	X_{21}, X_{22}
C_{15}	X_6
C_{16}	X_7
C_{17}	X_8, X_9

Table S2. The minimal path sets of hydraulic support failure

**Minimal path set**	**Basic event**
P_1	$X_1, X_2, X_6, X_7, X_8, X_{10}, X_{11}, X_{12}, X_{16}, X_{17}, X_{20}, X_{21}$
P_2	$X_1, X_2, X_6, X_7, X_8, X_{10}, X_{11}, X_{12}, X_{16}, X_{17}, X_{20}, X_{22}$
P_3	$X_1, X_2, X_6, X_7, X_8, X_{10}, X_{11}, X_{12}, X_{16}, X_{18}, X_{19}, X_{20}, X_{21}$
P_4	$X_1, X_2, X_6, X_7, X_8, X_{10}, X_{11}, X_{12}, X_{16}, X_{18}, X_{19}, X_{20}, X_{22}$
P_5	$X_1, X_2, X_6, X_7, X_8, X_{10}, X_{11}, X_{13}, X_{14}, X_{15}, X_{16}, X_{17}, X_{20}, X_{21}$
P_6	$X_1, X_2, X_6, X_7, X_8, X_{10}, X_{11}, X_{13}, X_{14}, X_{15}, X_{16}, X_{17}, X_{20}, X_{22}$
P_7	$X_1, X_2, X_6, X_7, X_8, X_{10}, X_{11}, X_{13}, X_{14}, X_{15}, X_{16}, X_{18}, X_{19}, X_{20}, X_{21}$
P_8	$X_1, X_2, X_6, X_7, X_8, X_{10}, X_{11}, X_{13}, X_{14}, X_{15}, X_{16}, X_{18}, X_{19}, X_{20}, X_{22}$
P_9	$X_1, X_2, X_6, X_7, X_9, X_{10}, X_{11}, X_{12}, X_{16}, X_{17}, X_{20}, X_{21}$
P_{10}	$X_1, X_2, X_6, X_7, X_9, X_{10}, X_{11}, X_{12}, X_{16}, X_{17}, X_{20}, X_{22}$
P_{11}	$X_1, X_2, X_6, X_7, X_9, X_{10}, X_{11}, X_{12}, X_{16}, X_{18}, X_{19}, X_{20}, X_{21}$
P_{12}	$X_1, X_2, X_6, X_7, X_9, X_{10}, X_{11}, X_{12}, X_{16}, X_{18}, X_{19}, X_{20}, X_{22}$
P_{13}	$X_1, X_2, X_6, X_7, X_9, X_{10}, X_{11}, X_{13}, X_{14}, X_{15}, X_{16}, X_{17}, X_{20}, X_{21}$
P_{14}	$X_1, X_2, X_6, X_7, X_9, X_{10}, X_{11}, X_{13}, X_{14}, X_{15}, X_{16}, X_{17}, X_{20}, X_{22}$
P_{15}	$X_1, X_2, X_6, X_7, X_9, X_{10}, X_{11}, X_{13}, X_{14}, X_{15}, X_{16}, X_{18}, X_{19}, X_{20}, X_{21}$
P_{16}	$X_1, X_2, X_6, X_7, X_9, X_{10}, X_{11}, X_{13}, X_{14}, X_{15}, X_{16}, X_{18}, X_{19}, X_{20}, X_{22}$
P_{17}	$X_1, X_3, X_4, X_5, X_6, X_7, X_8, X_{10}, X_{11}, X_{12}, X_{16}, X_{17}, X_{20}, X_{21}$
P_{18}	$X_1, X_3, X_4, X_5, X_6, X_7, X_8, X_{10}, X_{11}, X_{12}, X_{16}, X_{17}, X_{20}, X_{22}$
P_{19}	$X_1, X_3, X_4, X_5, X_6, X_7, X_8, X_{10}, X_{11}, X_{12}, X_{16}, X_{18}, X_{19}, X_{20}, X_{21}$
P_{20}	$X_1, X_3, X_4, X_5, X_6, X_7, X_8, X_{10}, X_{11}, X_{12}, X_{16}, X_{18}, X_{19}, X_{20}, X_{22}$
P_{21}	$X_1, X_3, X_4, X_5, X_6, X_7, X_8, X_{10}, X_{11}, X_{13}, X_{14}, X_{15}, X_{16}, X_{17}, X_{20}, X_{21}$
P_{22}	$X_1, X_3, X_4, X_5, X_6, X_7, X_8, X_{10}, X_{11}, X_{13}, X_{14}, X_{15}, X_{16}, X_{17}, X_{20}, X_{22}$
P_{23}	$X_1, X_3, X_4, X_5, X_6, X_7, X_8, X_{10}, X_{11}, X_{13}, X_{14}, X_{15}, X_{16}, X_{18}, X_{19}, X_{20}, X_{21}$
P_{24}	$X_1, X_3, X_4, X_5, X_6, X_7, X_8, X_{10}, X_{11}, X_{13}, X_{14}, X_{15}, X_{16}, X_{18}, X_{19}, X_{20}, X_{22}$
P_{25}	$X_1, X_3, X_4, X_5, X_6, X_7, X_9, X_{10}, X_{11}, X_{12}, X_{16}, X_{17}, X_{20}, X_{21}$
P_{26}	$X_1, X_3, X_4, X_5, X_6, X_7, X_9, X_{10}, X_{11}, X_{12}, X_{16}, X_{17}, X_{20}, X_{22}$
P_{27}	$X_1, X_3, X_4, X_5, X_6, X_7, X_9, X_{10}, X_{11}, X_{12}, X_{16}, X_{18}, X_{19}, X_{20}, X_{21}$
P_{28}	$X_1, X_3, X_4, X_5, X_6, X_7, X_9, X_{10}, X_{11}, X_{12}, X_{16}, X_{18}, X_{19}, X_{20}, X_{22}$
P_{29}	$X_1, X_3, X_4, X_5, X_6, X_7, X_9, X_{10}, X_{11}, X_{13}, X_{14}, X_{15}, X_{16}, X_{17}, X_{20}, X_{21}$
P_{30}	$X_1, X_3, X_4, X_5, X_6, X_7, X_9, X_{10}, X_{11}, X_{13}, X_{14}, X_{15}, X_{16}, X_{17}, X_{20}, X_{22}$
P_{31}	$X_1, X_3, X_4, X_5, X_6, X_7, X_9, X_{10}, X_{11}, X_{13}, X_{14}, X_{15}, X_{16}, X_{18}, X_{19}, X_{20}, X_{21}$
P_{32}	$X_1, X_3, X_4, X_5, X_6, X_7, X_9, X_{10}, X_{11}, X_{13}, X_{14}, X_{15}, X_{16}, X_{18}, X_{19}, X_{20}, X_{22}$
